# Plant pathogen nanodiagnostic techniques: forthcoming changes?

**DOI:** 10.1080/13102818.2014.960739

**Published:** 2014-10-22

**Authors:** Mohammad A. Khiyami, Hassan Almoammar, Yasser M. Awad, Mousa A. Alghuthaymi, Kamel A. Abd-Elsalam

**Affiliations:** ^a^ King Abdulaziz City for Science and Technology (KACST), Riyadh, Saudi Arabia; ^b^ Department of Agricultural Botany, Faculty of Agriculture, Suez Canal University, Ismailia, Egypt; ^c^ Biology Department, Science and Humanities College, Shaqra University, Alquwayiyah, Saudi Arabia; ^d^ Plant Pathology Research Institute, Agricultural Research Center (ARC), Giza, Egypt; ^e^ Unit of Excellence in Nano-Molecular Plant Pathology Research (ARC), Giza, Egypt

**Keywords:** diagnosis, quantum dots, nanobarcodes, nanosensory

## Abstract

Plant diseases are among the major factors limiting crop productivity. A first step towards managing a plant disease under greenhouse and field conditions is to correctly identify the pathogen. Current technologies, such as quantitative polymerase chain reaction (Q-PCR), require a relatively large amount of target tissue and rely on multiple assays to accurately identify distinct plant pathogens. The common disadvantage of the traditional diagnostic methods is that they are time consuming and lack high sensitivity. Consequently, developing low-cost methods to improve the accuracy and rapidity of plant pathogens diagnosis is needed. Nanotechnology, nano particles and quantum dots (QDs) have emerged as essential tools for fast detection of a particular biological marker with extreme accuracy. Biosensor, QDs, nanostructured platforms, nanoimaging and nanopore DNA sequencing tools have the potential to raise sensitivity, specificity and speed of the pathogen detection, facilitate high-throughput analysis, and to be used for high-quality monitoring and crop protection. Furthermore, nanodiagnostic kit equipment can easily and quickly detect potential serious plant pathogens, allowing experts to help farmers in the prevention of epidemic diseases. The current review deals with the application of nanotechnology for quicker, more cost-effective and precise diagnostic procedures of plant diseases. Such an accurate technology may help to design a proper integrated disease management system which may modify crop environments to adversely affect crop pathogens.

## Abbreviations

AuMNPs: magnetic gold nanoparticles
CdS: cadmium sulphide
CdTe: cadmium telluride
DON: deoxynivalenol
ELISA: enzyme-linked immunosorbent assay
FD: flavescence dorée
FSNP: fluorescent silica nanoparticles
LAMP: loop-mediated isothermal amplification
MRLs: maximum residue limits
NGS: next-generation sequence
OTA: ochratoxin A
QD: quantum dot
QPCR: quantitative polymerase chain reaction
RPA: polymerase Amplification reactions
SAED: selected-area electron diffraction
TEM: transmission electron microscopy
ZEA: zearalenone

## Introduction

Spread of plant diseases has internationally increased, while pathogen identification and control costs are still limited (i.e. ∼3% of the total costs of crop production).[1,2] Worldwide insect pests caused a 14% estimated loss, plant diseases caused a 13% loss and weeds caused a 13% loss. The value of this crop loss was assessed to be US $2000 billion per year.[3] Pathogens reduce plant growth and productivity due to generation of chronic stress conditions, and the methods for accurate disease diagnosis are expensive.[4] Several attempts have been conducted for safer crop production under different environments with protective devices or best management practices.[4] However, crop protection is the key to sustainable crop production, especially under adverse environmental conditions.

Traditional molecular diagnostic methods are widely used in laboratories all over the world to identify plant pathogenic organisms with high degree of sensitivity and specificity. Still, most of these procedures cannot be applied in the field (on-site detection) or in developing countries where incomes are small. Furthermore, the high price and short shelf half-life of some molecular biology reagents, such as enzymes and primers, limit the application of traditional molecular methods in developing countries. Nanotechnology may have actual solutions against many agriculture problems like plant disease control. Nano-based materials will be presented which will increase the efficacy of fungicides and pesticides, allowing only minor doses to be used.[5] Moreover, nanodiagnostic and microfluidics offer novel tools to improve the sample preparation step that remains difficult to integrate in a miniaturized platform. The signal amplification approaches could challenge those of target amplification. Quick on-site detection of plant pathogens using, nano-based kits, nanosenser, nanobiosensors, nanobarcodes and other portable diagnostic systems will also help the agricultural and food industry to manage different plant diseases. In this review, we describe the concepts and current state of the nanotechnology application in plant pathology including nanodiagnosis by portable polymerase chain reaction (PCR) systems, nanopore sequencing tools, nanodiagonastic kit, gold nanoparticles, quantum dots (QDs), nanobarcodes and nanosensors.

### Nanodiagnosis and nano-phytopathology

The extension/integration of molecular diagnostics on a nanoscale is a promising technology for identifying pathogens. Nanomolecular diagnostic is the use of nanobiotechnology to diagnose plant diseases and this can be termed as nanodiagnostics.[6] In particular, several nanodevices and nanosystems are used for sequencing single molecules of DNA. Assays with the use of nano-size devices to investigate DNA sequences and diagnose disease are becoming faster, more flexible and more sensitive.

It is noteworthy that new detection techniques involving nano-biosensors for pathogen identification will likely be a cornerstone to this trend.[7] During the 1980s and 1990s, phytopathologists relied on visual assessment to identify plant diseases.[3] Identifying plant pathogens via conventional techniques may take several days and therefore researchers need rapid detection tools that can provide results within a few hours. To develop such detection tools, phytopathologists are constantly working with nanotechnologists.[8] Nano and/or phytopathology researchers are attempting to develop an easy assay that is moveable and accurate and does not need any difficult method for procedure, so that farmers can use the mobile laboratory themselves to detect specific diseases.[9]

Newly developed nanomaterials with special nanoscale characteristics could present a tremendous breakthrough in pathogen and contaminant detection. Nanotechnology is also driving the development of lab-on-a-chip systems for detecting toxicity in waters, observing nutrients in irrigation water and controlling the quality in food production.[2,9]

**Figure 1.**
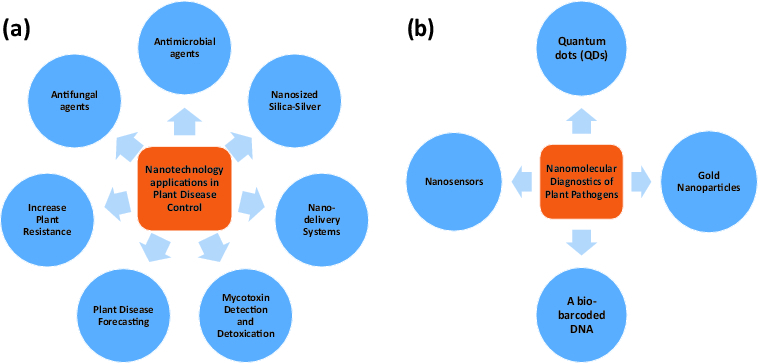
Potential nanotechnology applications in plant pathology: (a) plant disease control and (b) detection of plant pathogens.

Nano-phytopathology is a cutting-edge science which uses nanotechnology for detecting, diagnosing and controlling plant disease and their pathogens at an early stage, owing to crop protection from epidemic diseases. The modern plant pathologist strives to apply his knowledge in nanophytopathology to enable understanding of controlling factors of plant diseases and to develop and/or evaluate eco-friendly diagnostic measurements. Modern nanomolecular techniques are used for monitoring or understanding of pathogen population genetics, plant-microbe interactions and gene transfer between pathogens and even the host. Furthermore, nanoparticles such as nanosized silica-silver have recently been applied as antimicrobial and antifungal agents. Additionally, nanomaterials can be used for mycotoxin detection and detoxication, increasing plant resistance, plant disease forecasting and nano-molecular diagnostics of plant pathogens. Potential applications of nanophytopathology are shown in Figure 1.

The present review discusses the various applications of nanotechnology in plant pathogen detection.

## Plant pathogen diagnosis technologies

### Portable diagnostic equipment

#### Portable PCR systems

The capability to produce a fast PCR amplicon in a completely moveable set-up will not only allow plant pathologists to achieve more effective and predictable laboratory trials, but also considerably simplify other downstream applications such as those requiring in situ genomic tools for detection.

Palm PCR, developed by Ahram Biosystems Company in Korea, is a manageable thermal cycler designed for easy use, accuracy and high efficiency. Despite its tiny size, this controlling device delivers extremely well-organized and quick amplification for different types of DNA samples including the plant genome.[10] The DNA can be amplified in less than 25 minutes to enough quantity for ideal agarose-gel detection. The portable system presents a highly functional and user-friendly way to perform different types of PCR tests for both beginner and experienced researchers.

Compared to other diagnostic testing systems, Twista quantitative and portable real-time fluorometer is a customized device developed for examining recombinase polymerase amplification (RPA) reactions, and represents a new revolution in DNA diagnostics, as well as, combining advantage in speed, portability and ease of use with good sensitivity and specificity. Twista RPA fluorometer will supply immediate fast diagnosis, allowing an on-time suitable treatment compared with traditional microbiological assays, which require at least hours and molecular assays that typically need centralized equipment.

#### Portable genome sequencer (nanopore sequencing system)

**Figure 2.**
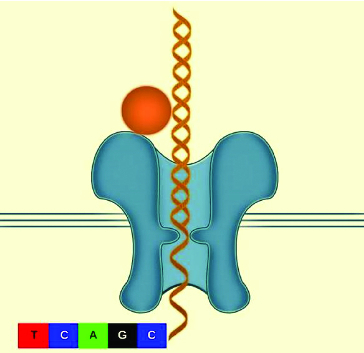
Diagram of a DNA molecule travelling through a protein nanopore.

Many companies are exploring the idea of using nanopore technology.[11,12] There are two main challenges that must be addressed in any nanopore sequencing: (1) how to distinguish the nucleotides as the strand passes through the nanopore and (2) how to control the speed of the DNA strand as it passes through the nanopore.[13] Even with these unique challenges the nanopore sequencing platform is thought to be simple and straightforward because theoretically very long reads can be generated from a low quantity of nucleic acid.[8,12] The protein nanopore and enzyme were designed to control a single strand of DNA, and as the DNA goes through the nanopore a direct electronic analysis is conducted. The protein nanopore is inserted in a polymer bilayer membrane across the top of a microwell. Each microwell has a sensor chip that measures the ionic current as the single molecule passes through the nanopore (Figure 2). However, the speed at which the DNA strand travels through the nanopore is too fast for accurate identification.[13] IBM and Roche together are developing a new sequencing technology described as ‘DNA transistor’ which could potentially record the nucleotide sequence as the template is pulled through the nanopore sensor.[14,15] One of the companies that is pioneering sequencing technologies, Oxford Nanopore Technologies, earlier this year announced that they expect nanopore strand sequencing to be able to produce a genomic map in 15 minutes at a cost of $1500 by 2014. Portable genome sequencer (MinION) was able to sequence 10 kb of a single sense and anti-sense DNA strand and will make next-generation sequence (NGS) within the reach of many groups and research environments. Nanopore platform implemented within current diagnostic equipment has the potential of analysing the entire genome in minutes instead of hours. Nanotechnology can be applied to plant science research in order to analyse plant genomics, gene function and pathogens detection as well as improvement of crop species.

#### Nanodiagonastic kit

Plant pathologists plan to study this ‘lab in a box’ very soon. This refers to packing sophisticated measuring devices, reagents, power supply and other features that now take up laboratory space into a parcel no larger or heavier than a briefcase.[16]

**Figure 3.**
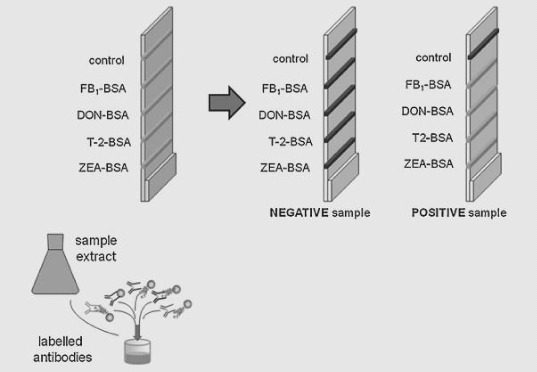
Schematic description of the 4mycosensor dipstick test (adapted from Lattanzio et al. [18]).

A briefcase-sized kit is transferred to a field where crops are growing to search for pathogens that could infect and reduce the yield. This is a quick and precise procedure. Nanodiagnostic kit equipment can easily and quickly detect potential serious plant pathogens, allowing experts to help farmers in prevention of disease epidemics from breaking out.[17] For example, 4mycosensor is a tetraplex competitive antibody-based assay in a dipstick format for the real-time detection of ZEA, T-2/HT-2, DON and FB1/FB2 mycotoxins on the same single strip in corn, wheat, oat and barley samples at or below their respective European maximum residue limits (MRLs).[18] A scheme of multiplex stripe used throughout the study (4mycosensor) is described in Figure 3. The proposed immunoassay protocol is fast, cheap, easy-to-use and suitable for the purpose of quick screening of mycotoxins in cereals.

#### Loop-mediated isothermal amplification (LAMP-PCR)

Loop-mediated isothermal amplification (LAMP) of DNA is a simple, cost effective, and rapid method for the specific on-site detection method of genomic DNA using a set of six oligonucleotide primers with eight binding sites hybridizing specifically to different regions of a target gene, and a thermophilic DNA polymerase from *Geobacillus stearothermophilus* for DNA amplification. There are several detection devices varying from gold nanoparticles tagged with short fragments of DNA to multicolour optical coding for biological tests that have been achieved by embedding different-sized QDs into polymeric microbeads.[6] The thiol-modified oligonucleotides (Au-nanoprobes) technique discards the use of electrophoretic analysis of LAMP-PCR products, thus increasing speed, specificity of results and decreasing costs.[19,20] LAMP has the potential to implement early detection of plant pathogens at a local level (e.g. on a farm) instead of in a laboratory or less well-resourced settings by molecular methods for monitoring inoculum levels in the air and is currently developing models to predict when and where a pathogen will first occur in the agricultural fields.

**Figure 4.**
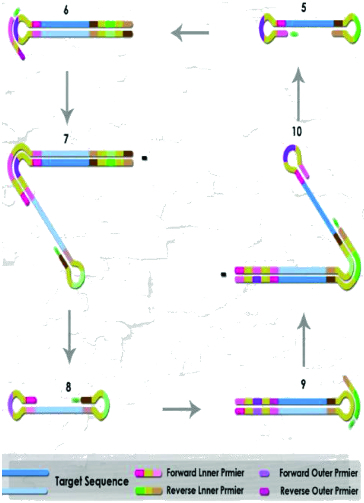
The LAMP-PCR cycle amplification step by Bst DNA polymerase with strand displacement activity.

For instance, LAMP-PCR of DNA is an easy, commercial and quick method for the specific detection of genomic DNA using a set of six oligo primers with eight binding sites hybridizing, specifically to various regions of a target gene (Figure 4), and a thermophilic DNA polymerase from *G. stearothermophilus* for DNA amplification of *Fusarium graminearum*.[21,22] Therefore, in the LAMP technique, diagnostics can be performed without examination of the amplification product during or after the amplification. Predominantly, positive reactions will be expected to show bright green fluorescence, while a negative reaction would remain light orange. This would be the expected result when SYBR Green I dye is added, meaning that the LAMP amplicons will be visualized directly and seen with the naked eye or under ultraviolet trans-illumination.

Positive results were obtained with *F. graminearum* isolates, while all reactions with other *Fusarium* and fungal species included for specificity remained negative, with a response identical to the negative control. This methodology is used to detect plant pathogenic fungi to rhizospheric microorganisms such as *Phytophthora sojae*,[23] *P. ramorum* and *P. kernoviae*,[24] *Aspergillus flavus* and *A. parasiticus*,[25] and toxigenic *Fusarium*.[21,22,26,27]

### Gold nanoparticles as biosensor

Utilization of nanomaterials or nanoparticles in biosensors allows the development and use of some novel signal detection procedures and equipment. Different strategies such as antibody–antigen, adhesion–receptor, antibiotic and complementary DNA sequence recognitions have been discovered for a specific detection between target phytopathogenic cells and bio-functionalized nanomaterials.[15,28]

Gold nanoparticles are excellent markers to be used in biosensors as several optical or electrochemical procedures can be modified to identify pathogens. A number of nanoparticle-based experiments have been performed to develop biomolecular detection with DNA- or protein-functionalized gold nanoparticles, which are used as the target-specific probes.[15,29] Several nanobiosensors for the molecular diagnosis of food-borne pathogens and agro-terrorism agents can be found in recent studies.[30] These detection methods include conductive polymer nanowires,[31] carbon nanotubes,[32] nanoporous silicon [30] and gold nanoparticles.[33] Dubertret et al. [34] highlighted the ability of gold nanoparticles to act as fluorescence quenchers and, therefore, it could be used to solve major drawbacks in molecular biology experiments. For example, a DNA oligonucleotide could be synthesized, fluorescently labelled at its 5′ end and conjugated at the 3′end with gold nanoparticles. These oligonucleotides can be used in diagnostic procedures, particularly in cases where DNA analysis cannot be circumvented, and in the diagnosis of the phytoplasma associated with the flavescence dorée (FD) of grapevine.[35] Fan et al. [15] reported that the gold nanoparticles efficiently quench the fluorescence of light harvester polymers, such as polyfluorene, and will open new perspectives in the development of the optical performances of nanobiotransducers for diagnostic purposes. Furthermore, a diagnostic probe made of a specific oligonucleotide bearing a fluorescein at its 5′ end and 2-nm gold particles at its 3′ end acts as a nanobiotransducer in DNA hybridization. It produces a stronger fluorescence signal when hybridized to target DNA.[35]

### Quantum dots (QDs)

QDs are semiconductor nanoparticles that fluoresce when stimulated by an excitation light source. Furthermore, QDs are inorganic fluorophores presenting major advantages over traditional organic fluorophores used as markers on nucleic acids or proteins for visual detection.[28,36] The mycosynthesis of semiconductor nanomaterials was first reported in unicellular yeast, which were shown to be capable of producing cadmium sulphide (CdS) crystallites in response to a cadmium salt stress.[37] Different microbes have also been used for the biosynthesis of CdS; however, few studies have focused on its luminescent properties. Very luminescent CdSe QDs were created by the fungus, *F. oxysporum* when incubated with a mixture of CdCl_2_ and SeCl_4_ at room temperature.[38] A proficient myco-mediated synthesis of highly fluorescent CdTe QDs was accomplished by the *F. oxysporum* isolates when reacted with a mixture of CdCl_2_ and TeCl_2_ at ambient conditions.[6] Description of these biosynthesized CdTe nanoparticles was performed by transmission electron microscopy (TEM) and selected-area electron diffraction (SAED).[39]

**Figure 5.**
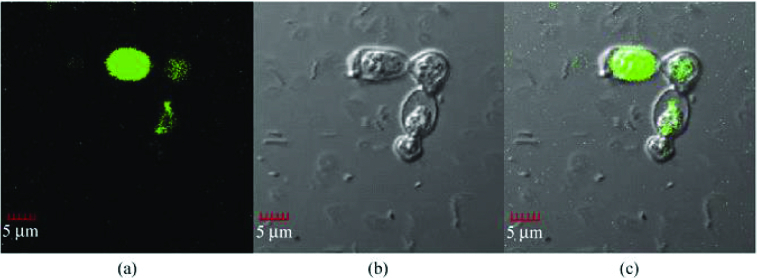
Confocal images of the yeast cells incorporated with the CdTe QDs at 35 °C for 8 days, recorded under excitation by a 488-nm laser giving green-emission (a), the bright field image (b) and the overlaid image (c) (adapted from Bao et al. [40]).

An effective biosynthesis method to prepare easily harvested biocompatible cadmium telluride (CdTe) QDs with tunable fluorescence emission using yeast cells was developed.[40] The confocal images of the yeast cells show obvious green emission from the synthesized CdTe QDs. The CdTe QD units were spread only in the cytoplasm and nucleus of yeast cells, while none were found in the cell membrane (Figure 5).

Biocompatible semiconductor crystals are composed of a nucleus and a shell allowing the binding of ligands and thus the attachment of this fluorescent marker to the target pathogen. A hybridization-based biosensor known as the quartz-crystal microbalance biosensor is a good example. The quartz-crystal microbalance vibrates under electric stimulus, and a change in mass due to the attachment of any compound to its surface can be detected through a reduction in the frequency of its vibration. When a nucleic acid probe is attached to the surface of a quartz-crystal microbalance biosensor and then exposed to complementary PCR product, a hybrid forms and causes the resonance frequency of the quartz-crystal microbalance biosensor to decrease dramatically. This system can be combined with fast PCR protocols to decrease the time for specific environmental detection of plant pathogens.[41]

### Nanobarcodes

#### A bio-barcoded DNA (b-DNA)

The bio-barcode assay is an ultrasensitive method of amplification and detection of proteins or nucleic acids. DNA bio-barcoded tests employ oligonucleotide-modified magnetic gold nanoparticles (AuMNPs) for signal amplification and for simple separation of a target protein from the sample. The large b-DNA-to-recognition agent ratio affords a means of substantial signal amplification. It is also promising by allowing the quick detection of numerous protein targets at low-attomolar concentrations [16] and nucleic acids at high-zeptomolar levels under optimized conditions.[42] The concept of the bio-barcode assay is unique and represents a potential alternative to the PCR technique.

#### Nanostructured platform for mycotoxin detection

Nanostructured platform for mycotoxin detection and detoxification mycotoxins are secondary metabolites produced by pathogenic filamentous fungi causing disease and death in humans and other animals. Mycotoxicoses are diseases caused by the ingestion of mycotoxins by humans and animals, mostly through the consumption of polluted food. They can induce various different biological effects, i.e. carcinogenic, mutagenic, teratogenic, estrogenic, immunotoxic, nephratoxic and neurotoxic effects.[43,44]

A variety of biosensors have been developed and described in the recent literature for mycotoxin analysis some of which have the potential of multi-array mycotoxins identification. Prieto-Simon et al. [45] reviewed emerging biotechnological methods for mycotoxin analysis and confirmed that nanotechnology has recently been incorporated into mycotoxin bioassays. The main role of nanosensors is to decrease the time for fungal pathogen detection.[46] Food spoilage can be detected by nanosensors such as an array of thousands of nanoparticles designed to be visualized in different colours in contact with food pathogens.[47]

Nanosensors could be placed directly into the packaging material, where they would serve as ‘electronic noses’ which can detect chemicals released during food spoilage. Some nanosensors are based on microfluidic devices,[46] and they can be used to identify pathogens proficiently in short time with high sensitivity. Sol–gel derived nano-ZnO coat can be used for the immobilization of r-IgGs, while bovine serum albumin (BSA) can be used for blocking non-specific binding sites of r-IgGs to identify ochratoxin A (OTA). BSA/r-IgGs/nano-ZnO/ITO platform for detection of mycotoxins such as aflatoxins (APH), ochratoxin B, citrinin, patulin, ergot akaloids, fumonisins, trichothecenes and zearalenone (ZEA) is still under development.[48] A novel ultra-sensitive magnetic nanoparticle immunoassay for mycotoxin detection was developed to provide real-time quantitative results for detecting more than one mycotoxin.[49] Moreover, real-time assays were performed upon the addition of magnetic nanotags onto the spin-valve sensor surface immobilized with capture antibodies for detection of mycotoxins (aflatoxin-B1, zearalenone and HT-2). Lattanzio et al. described a new method, signal transduction by ion nano-gating sensors which are used as an analytical technique for the ultrasensitive detection of mycotoxins, with a detection limit up to 100 fg/mL.[50]

An immunosensor technique coupled with a flow injection system can be used for the quick, sensitive and discriminatory quantification of zearalenol in corn silage samples.[51] Horseradish peroxidase (HRP) biosensors have been applied without any pre-treatment to determine OTA in spiked beer samples, and OTA isolated from roasted coffee. Additionally, the performance of the HRP biosensors has been revealed yielding average revivals of 103% and 99%, respectively in both cases of OTA as mentioned earlier.[52] The ozonization and adsorption efficiency of modified nanodiamonds to decrease the content of aflatoxin-B1 has been examined by Puzyr et al. [53]. Silica and clays are most efficient in combination with smaller sized water molecules and smaller mycotoxins such as aflatoxins and ochratoxins. However, clays are less efficient in binding the larger mycotoxins such as fumonisin and deoxynivalenol (vomitoxin) because the distance among clay layers is not sufficient to accommodate the larger molecules.[54] By using nano-sized clay, the space between the layers of clay has been prolonged 10 times. As a result, the nanoclay can bind the whole family of mycotoxins. A rapid enzyme-linked immunosorbent assay (ELISA) method was investigated by reducing the coating, blocking and competition time required in usual ELISA method using superparamagnetic nanoparticles.[55] This method was effective for detecting aflatoxin M1 (AFM1) in milk in a linear working range of 4–250 ng/L.[55] A nanostructured cerium oxide film-based immunosensor was also developed for the detection of food-borne mycotoxins. Rabbit-immunoglobulin antibodies and BSA have been immobilized onto sol–gel-derived nanostructured cerium oxide film synthesized onto an indium tin-oxide covered glass plate for the detection of ochratoxin-A.[56,57] Recently, Paniel et al. [44] developed an electrochemical immunosensor for the detection of ultra-trace quantities of AFM1 in foodstuffs. These immunosensors were prepared by using magnetic nanoparticles and a competitive immunoassay using HRP as a label and were able to detect small amounts of AFM1 (up to 0.01 ppb). Cysteamine functionalized-gold nanoparticles (C-AuNP) along with covalently attached aflatoxin B1 antibodies (aAFB1) were immobilized onto a 4-mercaptobenzoic acid-based self-collected monolayer on a gold electrode (MBA/Au) to prepare a BSA/aAFB1-C-AuNP/MBA/Au immunoelectrode. These electrodes were used to detect AFB1 in the range of 10–100 ng/L.[58] A moveable machine has recently been developed that can concurrently identify various bacterial, fungal toxins and pathogens in stored food. Obviously biosensors can be an exciting alternative to the conventional techniques for the detection of mycotoxins and pathogens in food.[7,13,59]

### Nanosensors

#### Nanosensors and plant disease predicting

Plant disease forecasting is a management method used to forecast the chances or severity of plant diseases and to help farmers make cost-effective decisions for controlling diseases.[60] Presently research is being carried out by using nanosensors to improve pathogen detection methods in crop systems.[8,61] Many electronic companies have been examining the use of electrical conducting polymers such as polyaniline, polythiophene and polypyrolle. These polymers can also be used to fabricate sensors that can detect molecular signals with very low intensity of spoilage and food-borne pathogens within minutes.[62] The bio-nanosensor has the potential of increased sensitivity and therefore a significantly reduced response-time to discover potential disease problems. Such bio-analytical nanosensors were utilized to detect and quantify minute amounts of contaminants such as viruses bacteria, fungi, toxins and other bio-hazardous substances in the agriculture and food systems. Therefore, these biosensors may have a huge impact on the precision farming methods.[8]

Nano-sensors can be linked to a GPS for real-time monitoring of disease and distributed throughout the field to monitor soil conditions and crop health.[8,13] The union of biotechnological and nanotechnological approaches in bio-sensors will be used to construct equipment with increased sensitivity, allowing an earlier response to ecological changes and disease prevalence.[8] Nanosensors will allow us to identify plant diseases before visible symptoms appear and thus facilitate their control. Precision farming will allow improved agriculture production by providing precise data, helping growers to make better decisions.[8]

#### Nanobiosensors in agriculture

The nanobiosensor is the product of a combined approach of biology and nanotechnology.[30] These sensors hold the potential of increased sensitivity and therefore a significantly reduced response-time to sense potential disease problems in crops [63] and thus they can help enhance production and improve food safety in agriculture. To detect pathogens and toxins in food, fibre-optic biosensor has commonly used tapered fibre-optic probes coated with antibodies. An approach has been developed for rapid determination of *Escherichia coli* using a flow-injection system. Electrochemical measurement of K_3_Fe (CN)_6_, reduced by microbial metabolism, allowed the quantitative determination of bacteria and fungi in 20 minutes. Hashimoto et al. [64] developed a new biosensor system for the rapid diagnosis of soil-borne diseases, consisting of two biosensors. The system was constructed using equal quantities of two different microbes, each individually immobilized on an electrode.

Taking into consideration the particular optical properties of silver nanoparticles, the interaction between silver nanoparticles and sulphurazon-ethyl herbicide was investigated.[65] They found that silver nanoparticles are sensitive to increased concentrations of herbicide in a solution and induced a variation in colour of the nanoparticles from yellow to orange red and finally to purple. This approach is useful for detecting contaminants, such as organic pollutants and microbial pathogens in water bodies and in the environment.[34] Fluorescent silica nanoparticles (FSNP) combined with antibody molecules successfully detected plant pathogens such as *Xanthomonas axonopodis* pv. *vesicatoria* which causes bacterial spot disease in tomatoes and peppers.[66] Copper oxide (CuO) nanoparticles and nanolayers were synthesized by sol–gel and spray pyrolysis methods, respectively. Both CuO nanoparticles and nanostructural layer biosensors were used for detecting the *A. niger* fungi.[67]

### Nanofabrication imaging

Diagnostic imaging refers to a broad slew of technologies used to look inside or outside plant tissues in order to diagnose various plant pathogens. The rapidly expanding use of diagnostic imaging technologies continues to push the boundaries of plant pathologist diagnostic capabilities. Using these techniques, plant doctors are able to diagnose crop diseases earlier and more precisely.[68] Nanotechnology offers us the opportunity to precisely tune and control the chemical and physical properties of contrast materials in order to overcome concerns with toxicity, useful imaging time, tissue specificity and signal strength. In the ‘mesoscopic’ size range of 5–100 nm diameter, nanoparticles also have large surface areas and functional groups for conjugating to multiple diagnosis tools.[69] Thus, progress in the field of nano-scale contrast agents will play a key role in the continued enhancement of our diagnostic imaging capabilities in the coming years.

**Figure 6.**
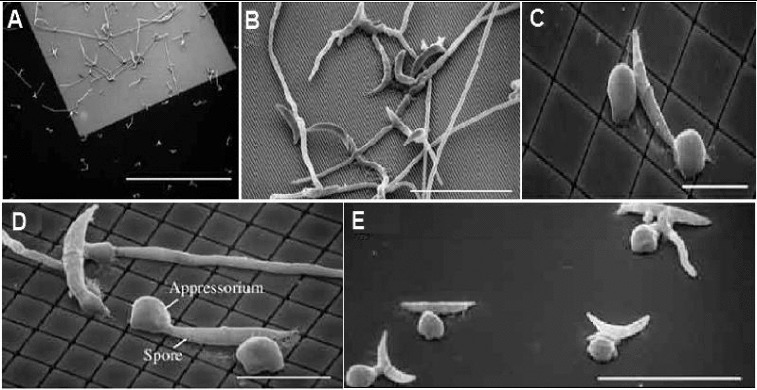
Scanning electron micrographs (SEM) showing the fungus *Colletotrichum graminicola* grown on nanofabricated pillared arrays. When the individual pillars are very small (0.5 μm wide) and do not provide much surface contact (A, B), the spores of the fungus grow without forming ‘appressoria’. When the pillars are wider (C, D) or when the surface is completely smooth (E), appressoria are formed quickly. Scale bars, 500, 50, 20, 20, and 50 μm, respectively (adopted from Mccandless [70]).

For example, electron beam and photolithography techniques were used to fabricate topographies that mimic leaf surface features as well as the internal plumbing of plants, and then nano-imaging technologies were used to study how bacteria and fungi invade and colonize the leaf.[70] Lithography was used to nanofabricate a pillared surface on silicon wafers. This lawn of miniature pillars was between 1.4 and 20 μm wide and spaced various distances apart. It was used to examine the movement across the surface by the fungus that mimicked some of the characteristics of the host plant. Images of the *Colletotrichum graminicola* crawling across the nanofabricated surface assisted the researchers to determine that the fungus needs to make a minimum contact of at least 4.5 μm before it starts to develop appressoria (Figure 6). To develop disease resistant cultivars, the infection process and behaviour of bacterium pathogen causing Pierce's disease inside grapevine xylem were studied using nanofabrication methods.[71]

The application of carbon-coated magnetic nanoparticles and microscopy methods at different levels of resolution to visualize and path the transport and deposition of nanoparticles inside the plant host was reported by González-Melendi et al. [72].

## Conclusion

The portable diagnostic equipment, nanoparticle-based, bio-barcoded DNA sensor, and the QD have potential applications in the multiple detection of plant pathogens and toxigenic fungi. To date, mobile diagnostic assays have been developed to rapidly detect plant disease and may be used to prevent epidemics. These nano-based diagnostic kits not only increase the speed of pathogen detection but also increase the accuracy of the diagnosis. Additionally, the combination of nanotechnology with microfluidic systems has been effectively applied in molecular plant pathology and can be adapted to detect specific pathogens and toxins. A good example is the micro-PCR where 40 cycles of PCR can be performed in less than 6 minutes. In the near future, nano-scale devices with novel properties could be used to make smart agricultural systems. For instance, these nanodevices could be used to identify plant health issues before these become observable to the grower. Such devices may be capable of responding to special situations, identifying the problem and taking an appropriate disease management action. In this way, nanosmart devices will act as both a protective and an early warning system. During the next decade, nanodevices, which can make thousands of measurements speedily and very cheaply, will become available. Future prospects in plant disease diagnostic will continue in miniaturization of biochip technology to the nanoscale range. Nanophytopathology can be applied as a tool to understand plant–pathogen interactions, providing new methods for crop protection. Specific nanodevices and DNA nanodevices could enable accurate tracking, detection and diagnosis of plant pathogens in the early stages of plant disease.

## Application prospects

1. Quick response within integrated disease management system via external and implanted nanosensor systems.
2. Improvement of rapid laboratory biosensors to detect plant pathogens in the field or post-harvest.
3. Development of retrieval nanosystems for a specific sampling from soil, air and plant samples.
4. Provide rapid and reliable NANO methods for detection of mycotoxins and toxigenic fungi.
5. Detection of pesticide residues in food and feeds.

## References

[cit0001] Agrios GN (2005). Plant pathology.

[cit0002] van Lenteren JC, Dent D (1995). Integrated pest management in protected crops. Integrated pest management.

[cit0003] Lenteren JC van, Martin NA, Albajes R, Gullino ML, van Lenteren JC, Elad Y (1999). Biological control of whitefly.

[cit0004] Leake JR, Donnelly DP, Boddy L, Van der Heijden MGA, Sanders IR (2002). Interactions between ecto-mycorrhizal fungi and saprotrophic fungi. Mycorrhizal ecology.

[cit0005] Abd-Elsalam KA ( 2013). Nanoplatforms for plant pathogenic fungi management. Fungal Genome Biol..

[cit0006] Jain K. (2003). Nanodiagnostics: application of Nanotechnology (NT) in molecular diagnostics. Expert Rev Mol Diagn.

[cit0007] Yalcin B, Otles S (2010). Nanobiosensor and food pathogen interaction mechanisms. Electron J Environ Agric Food Chem..

[cit0008] Rai M, Ingle A (2012). Role of nanotechnology in agriculture with special reference to management of insect pests. Appl Microbiol Biotechnol..

[cit0009] Gardeniers JG, van den Berg AA (2004). Lab-on-a-chip systems for biomedical and environmental monitoring. Anal Bioanal Chem..

[cit0010] Monis PT, Giglio S (2006). Nucleic acid amplification-based techniques for pathogen detection and identification. Infect Genet Evol..

[cit0011] Niedringhaus TP, Milanova D, Kerby MB, Snyder MP, Barron AE (2011). Landscape of next-generation sequencing technologies. Anal Chem..

[cit0012] Ozsolak F (2012). Third-generation sequencing techniques and applications to *drug discovery*. Expert Opin Drug Discover..

[cit0013] Clarke J, Wu H-C, Jayasinghe L, Patel A, Reid S, Bayley H (2009). Continuous base identification for single-molecule nanopore DNA sequencing. Nat Nanotechnol..

[cit0014] Zhang J, Chiodini R, Badr A, Zhang GF (2011). The impact of next-generation sequencing on genomics. J Genet Genomics..

[cit0015] Fan C, Wang S, Hong JW, Bazan GC, Plaxco KW, Heeger AJ (2003). Beyond superquenching: hyper-efficient energy transfer from conjugated polymers to gold nanoparticles. Proc Natl Acad Sci..

[cit0016] Goluch ED, Nam JM, Georganopoulou DG, Chiesl TN, Shaikh KA, Ryu KS, Barron AE, Mirkin CA, Liu C (2006). A bio-barcode assay for on-chip attomolar-sensitivity protein detection. Lab Chip..

[cit0017] Pimentel D, Inderjit (2009). Invasive plants: their role in species extinctions and economic losses to agriculture in the USA. Management of invasive weeds, invading nature – Springer Series in invasion ecology.

[cit0018] Lattanzio VMT, Nivarlet N, Lippolis V, Gatta SD, Huet A-C, Delahaut P, Granier B, Visconti A (2012). Multiplex dipstick immunoassay for semi-quantitative determination of Fusarium mycotoxins in cereals. Anal Chim Acta.

[cit0019] Kaewphinit T, Santiwatanakul S, Chansiri K (2013). Colorimetric DNA based biosensor combined with loop-mediated isothermal amplification for detection of mycobacterium tuberculosis by using gold nanoprobe aggregation. Sens Transducers..

[cit0020] Zhou C, Mu Y, Yang M, Wu Q, Xu W, Zhang Y, Jin W, Song Q, Wu Z, Jin Q (2013). Gold nanoparticles based colorimetric detection of target DNA after loop-mediated isothermal amplification. Chem Res Chin Univ..

[cit0021] Abd-Elsalam KA,, Bahkali A, Moslem M,, Amin O, Niessen L (2011). An optimized protocol for DNA extraction from wheat seeds and loop-mediated isothermal amplification (LAMP) to detect *Fusarium graminearum* contamination of wheat grain. Int J Mol Sci..

[cit0022] Niessen L, Vogel RF (2010). Detection of *Fusarium graminearum* DNA using a loop-mediated isothermal amplification (LAMP) assay. Int J Food Microbiol..

[cit0023] Dai TT, Lu CC, Lu J, Dong S, Ye W, Wang Y, Zheng X (2012). Development of a loop-mediated isothermal amplification assay for detection of *Phytophthora sojae*. FEMS Microbiol Lett..

[cit0024] Tomlinson JA, Dickinson MJ, Boonham N (2010). Rapid detection of *Phytophthora ramorum* and *P. kernoviae* by two-minute DNA extraction followed by isothermal amplification and amplicon detection by generic lateral flow device. Phytopathology..

[cit0025] Luo J, Vogel RF, Niessen L (2012). Development and application of a loop-mediated isothermal amplification assay for rapid identification of aflatoxigenic molds and their detection in food samples. Int J Food Microbiol..

[cit0026] Niessen L, Gräfenhan T, Vogel RF (2012). ATP citrate lyase 1 (acl1) gene-based loop-mediated amplification assay for the detection of the *Fusarium tricinctum* species complex in pure cultures and in cereal samples. Int J Food Microbiol..

[cit0027] Denschlag C, Vogel RF, Niessen L (2012). Hyd5 gene-based detection of the major gushing-inducing *Fusarium* spp. in a loop-mediated isothermal amplification (LAMP) assay. Int J Food Microbiol..

[cit0028] Wang L, O’Donoghue MB, Tan W (2006). Nanoparticles for multiplex diagnostics and imaging. Nanomedicine..

[cit0029] Thaxton CS, Georganopoulou DG, Mirkin CA (2006). Gold nanoparticle probes for the detection of nucleic acid targets. Clin Chim Acta..

[cit0030] Yang H, Li H, Jiang X (2008). Detection of foodborne pathogens using bioconjugated nanomaterials. Microfluid Nanofluid..

[cit0031] Pal S, Ying W, Alocilja EC, Downes FP (2008). Sensitivity and specificity performance of a direct-charge transfer biosensor for detecting *Bacillus cereus* in selected food matrices. Biosyst Eng..

[cit0032] Poonam P, Deo N (2008). Current correlation functions for chemical sensors based on DNA decorated carbon nanotube. N Sens Actuators B: Chem.

[cit0033] Wang J, Zhang S, Zhang Y (2010). Fabrication of chronocoulometric DNA sensor based on gold nanoparticles/poly(l-lysine) modified glassy carbon electrode.

[cit0034] Dubertret B, Calame M, Libchaber AJ (2001). Single-mismatch detection using gold-quenched fluorescent oligonucleotides. Nat Biotechnol..

[cit0035] Firrao G, Moretti M, Ruiz-Rosquete M, Gobbi E, Locci R (2005). Nanobiotransducer for detecting flavescence dorée phytoplasma. J Plant Pathol..

[cit0036] Arya H, Kaul Z, Wadhwa R, Taira K, Hirano T, Kaul SC (2005). Quantum dots in bio-imaging: revolution by the small. Biochem Biophys Res Commun..

[cit0037] Dameron CT, Reeser RN, Mehra RK, Kortan AR, Carroll PJ, Steigerwaldm ML, Brus LE, Winge DR (1989). Biosynthesis of cadmium sulphide quantum semiconductor crystallites. Nature.

[cit0038] Kumar S, Ansary A, Ayoobul A, Absar A, Khan MI (2007). Extracellular biosynthesis of CdSe quantum dots by the fungus, *Fusarium oxysporum*. J Biomed Nanotechnol..

[cit0039] Syed A,, Ahmad A (2013). Extracellular biosynthesis of CdTe quantum dots by the fungus *Fusarium oxysporum* and their anti-bacterial activity. Spectrochim Acta Part A: Mol Biomol Spectrosc.

[cit0040] Bao H, Hao N, Yang Y, Zhao D (2010). Biosynthesis of biocompatible cadmium telluride quantum dots using yeast cells. Nano Res..

[cit0041] Sharon M, Choudhary AK, Kumar RJ (2010). Nanotechnology in agricultural diseases and food safety. J Phytol..

[cit0042] Nam JM, Stoeva SI, Mirkin CA (2004). Bio-bar-code-based DNA detection with PCR-like sensitivity. J Am Chem Soc.

[cit0043] Kankkunen P, Rintahaka J, Aalto A, Leino M, Majuri ML, Alenius H, Wolff H, Matikainen S (2009). Trichothecene mycotoxins activate inflammatory response in human macrophages. J Immunol..

[cit0044] Paniel N, Radoi A, Marty J-L (2010). Development of an electrochemical biosensor for the detection of aflatoxin M1 in milk. Sensors..

[cit0045] Prieto-Simon B, Noguer T, Campas M (2007). Emerging biotools for assessment of mycotoxins in the past decade. Trends Anal Chem..

[cit0046] Baeummer A (2004). Nanosensors identify pathogens in food. Food Technol..

[cit0047] Bhattacharya S, Jang J, Yang L, Akin D, Bashir R (2007). Biomems and nanotechnology based approaches for rapid detection of biological entities. J Rapid Methods Automation Microbiol.

[cit0048] Ansari AA, Kaushik A, Pratima R, Solanki Malhotra BD (2010). Nanostructured zinc oxide platform for mycotoxin detection. Bioelectrochemistry.

[cit0049] Mak AC, Osterfeld SJ, Yu H, Wang SX, Davis RW, Jejelowo OA, Pourmand N (2010). Sensitive giant magnetoresistive-based immunoassay for multiplex mycotoxin detection. Biosens Bioelectron..

[cit0050] Actis P, Jejelowo O, Pourmand N (2010). Ultrasensitive mycotoxin detection by STING sensors. Biosens Bioelectron..

[cit0051] Panini NV, Bertolino FA, Salinas E, Messina GA, Raba J (2010). Zearalenone determination in corn silage samples using an immunosensor in a continuous-flow/stopped-flow systems. Biochem Eng J..

[cit0052] Alonso-Lomilloa MA, Domínguez-Renedoa O, Ferreira-Gonc L, Arcos-Martíneza MJ (2010). Sensitive enzyme-biosensor based on screen-printed electrodes for ochratoxin A. Biosens Bioelectron..

[cit0053] Puzyr AP, Burov AE, Bondar VS, Trusov YN (2010). Neutralization of aflatoxin b1 by ozone treatment and adsorption by nanodiamonds. Nanotechnol Russian.

[cit0054] Jaynes WF, Zartman RE, Hudnall WH (2007). Aflatoxin B1 adsorption by clays from water and corn meal. Appl Clay Sci..

[cit0055] Radoi A, Targa M, Prieto-Simon B, Marty JL (2008). Enzyme-linked … nanoparticles for aflatoxin M1 detection. Talanta..

[cit0056] Kaushik A, Solanki PR, Ansari AA, Ahmad S, Malhotra BS (2009). A nanostructured cerium oxide film-based immunosensor for mycotoxin detection. Nanotechnology.

[cit0057] Kaushik A, Arya SK, Vasudev A, Bhansali S (2013). Open J Appl Biosens..

[cit0058] Sharma A, Matharu Z, Sumana G, Solanki PR, Kim CG, Malhotra BD (2010). Antibody immobilized cysteamine functionalized-gold nanoparticles for aflatoxin detection. Thin Solid Films..

[cit0059] Biswal SK, Nayak AK, Parida UK, Nayak PL (2012). Applications of nanotechnology in agriculture and food sciences. Int J Sci Innovations Discov..

[cit0060] Bogue B (2008). Nanosensors: a review of recent progress.

[cit0061] Esker PD, Sparks AH, Campbell L, Guo Z, Rouse M, Silwal SD, Tolos S, Van Allen B, Garrett KA (2008). Ecology and epidemiology in R: disease forecasting and validation. [Online]. Plant Health Instructor..

[cit0062] Sekhon BS (2010). Food nanotechnology – an overview. J Nanotechnol Sci Appl..

[cit0063] Small J, Call DR, Brockman FJ, Straub TM, Chandler DP (2001). Direct detection of 16S rRNA in soil extracts by using oligonucleotide microarrays. Appl Environ Microbiol..

[cit0064] Hashimoto Y, Nakamura H, Koichi AK, Karube I (2008). A new diagnostic method for soil-borne disease using a microbial biosensor. Microbes Environ..

[cit0065] Dubas ST, Pimpan V (2008). Green synthesis of silver nanoparticles for ammonia sensing. Talanta..

[cit0066] Yao KS, Li SJ, Tzeng KC, Cheng TC, Chang CY, Chiu CY, Liao CY, Hsu JJ, Lin ZP (2009). Fluorescence silica nanoprobe as a biomarker for rapid detection of plant pathogens.. Multi-Funct Mater Struct II. Parts 1 and 2.

[cit0067] Etefagh R, Azhir E, Shahtahmasebi N (2013). Synthesis of CuO nanoparticles and fabrication of nanostructural layer biosensors for detecting *Aspergillus niger* fungi. Sci Iranian..

[cit0068] Rosen JE, Yoffe S, Meerasa A, Verma M, Gu FX (2011). Nanotechnology and diagnostic imaging: new advances in contrast agent technology. J Nanomed Nanotechnol..

[cit0069] Nie L ( 2013). Biomedical nanotechnology for optical molecular imaging, diagnostics, and therapeutics. JSM Nanotechnol Nanomed..

[cit0070] Mccandless L (2005). Nanotechnology offers new insights into plant pathology.

[cit0071] Meng Y, Li Y, Galvani CD, Hao G, Turner JN, Burr TJ, Hoch HC (2005). Upstream migration of *Xylella fastidiosa* via Pilus-Driven twitching motility. J Bacteriol..

[cit0072] González-Melendi P, Fernandez-Pacheco R, Coronado MJ, Corredor E, Testillano PS, Risueño MC, Marquina C, Ibarra MR, Rubiales D, Pérez-de-Luque A (2007). Nanoparticles as smart treatment delivery systems in plants: assessment of different techniques of microscopy for their visualisation in plant tissues. Ann Bot..

